# The Overlapping Area of Non-Celiac Gluten Sensitivity (NCGS) and Wheat-Sensitive Irritable Bowel Syndrome (IBS): An Update

**DOI:** 10.3390/nu9111268

**Published:** 2017-11-21

**Authors:** Carlo Catassi, Armin Alaedini, Christian Bojarski, Bruno Bonaz, Gerd Bouma, Antonio Carroccio, Gemma Castillejo, Laura De Magistris, Walburga Dieterich, Diana Di Liberto, Luca Elli, Alessio Fasano, Marios Hadjivassiliou, Matthew Kurien, Elena Lionetti, Chris J. Mulder, Kamran Rostami, Anna Sapone, Katharina Scherf, Detlef Schuppan, Nick Trott, Umberto Volta, Victor Zevallos, Yurdagül Zopf, David S. Sanders

**Affiliations:** 1 Department of Pediatrics, Marche Polytechnic University, 60121 Ancona, Italy; c.catassi@univpm.it (C.C.); mariaelenalionetti@gmail.com (E.L.); 2 Department of Medicine, Columbia University Medical Center, New York, NY 10027, USA; aa819@cumc.columbia.edu; 3 Medical Department, Division of Gastroenterology, Infectiology and Rheumatology, Charité, Campus Benjamin Franklin, 12203 Berlin, Germany; christian.bojarski@charite.de; 4 Department of Gastroenterology and Liver Diseases, CHU, 38043 Grenoble, France; bbonaz@chu-grenoble.fr; 5 Celiac Center Amsterdam, Department of Gastroenterology, VU University Medical Center, 1117 Amsterdam, The Netherlands; g.bouma@vumc.nl (G.B.); cjmulder@vumc.nl (C.J.M.); 6 Department of Internal Medicine, “Giovanni Paolo II” Hospital, Sciacca (AG) and University of Palermo, 92019 Sciacca, Italy; acarroccio@hotmail.com; 7 Paediatric Gastroenterology Unit, Sant Joan de Reus University Hospital. IISPV, 43003 Tarragona, Spain; gcv@tinet.cat; 8 Department of Internal and Experimental Medicine Magrassi-Lanzara, University of Campania Luigi Vanvitelli, 80131 Naples, Italy; laura.demagistris@unicampania.it; 9 Medical Clinic 1, Friedrich-Alexander-University Erlangen-Nürnberg, 91054 Erlangen, Germany; walburga.dieterich@uk-erlangen.de (W.D.); Yurdaguel.Zopf@uk-erlangen.de (Y.Z.); 10 Central Laboratory of Advanced Diagnosis and Biomedical Research (CLADIBIOR), University of Palermo, 90133 Palermo, Italy; diana.diliberto@unipa.it; 11 Center for the Prevention and Diagnosis of Celiac Disease, Fondazione IRCCS Ca Granda Ospedale Maggiore Policlinico, 20122 Milan, Italy; lucelli@yahoo.com; 12 Center for Celiac Research and Treatment, Massachusetts General Hospital, Boston, MA 02114, USA; AFASANO@mgh.harvard.edu (A.F.); annasapone@yahoo.it (A.S.); 13 Academic Department of Neurosciences, Sheffield Teaching Hospitals NHS Foundation Trust, Sheffield S10 2JF, UK; Marios.Hadjivassiliou@sth.nhs.uk; 14 Academic Unit of Gastroenterology, Department of Infection, Immunity & Cardiovascular Disease, University of Sheffield, Sheffield S10 2TN, UK; matthew.kurien@sth.nhs.uk (M.K.); nick.trott@sth.nhs.uk (N.T.); 15 Gastroenterology Unit, Milton Keynes University Hospital, Milton Keynes MK6 5LD, UK; krostami@hotmail.com; 16 German Research Centre for Food Chemistry, Leibniz Institute, Lise-Meitner-Straße 34, D-85354 Freising, Germany; Katharina.Scherf@lrz.tu-muenchen.de; 17 Institute of Translational Immunology, University Medical Center, Johannes Gutenberg University, 55131 Mainz, Germany; detlef.schuppan@unimedizin-mainz.de (D.S.); zevallos@uni-mainz.de (V.Z.); 18 Department of Medical and Surgical Sciences, University of Bologna, 40138 Bologna, Italy; umberto.volta@aosp.bo.it

**Keywords:** gluten sensitivity, celiac disease, wheat allergy, gluten-related disorders, gluten-free diet, amylase-trypsin inhibitors (ATIs)

## Abstract

Gluten-related disorders have recently been reclassified with an emerging scientific literature supporting the concept of non-celiac gluten sensitivity (NCGS). New research has specifically addressed prevalence, immune mechanisms, the recognition of non-immunoglobulin E (non-IgE) wheat allergy and overlap of NCGS with irritable bowel syndrome (IBS)-type symptoms. This review article will provide clinicians with an update that directly impacts on the management of a subgroup of their IBS patients whose symptoms are triggered by wheat ingestion.

## 1. Introduction

Non-celiac gluten sensitivity (NCGS), sometimes referred as gluten sensitivity, gluten intolerance, or non-celiac wheat sensitivity, was already described in 1978 but did not receive much recognition from clinicians until the 21st century [1]. It is characterized by intestinal and extra-intestinal symptoms related to the ingestion of gluten-containing food, in subjects that are not affected by either celiac disease (CD) or wheat allergy (WA). This is the original definition based on the Salerno Experts’ Criteria [2]. However, our understanding has evolved since that time and we recognized that the spectrum of symptoms, which occur maybe due not just to the ingestion of gluten proteins but potentially other wheat-related components. In clinical practice, it is crucial to exclude CD in patients who present in this way. A recent PubMed search using the Medical Subject Headings (MeSH) index term of NCGS demonstrates 125 publications prior to 2000 but 490 since the start of the new millennium. Public and commercial interest in a gluten free diet (GFD) has galvanized the scientific community into undertaking research beyond the previously recognized gluten-related disorders such as celiac disease or dermatitis herpetiformis.

Irritable bowel syndrome (IBS) is a common intestinal disorder causing abdominal pain, bloating, gas, diarrhea and constipation. IBS-like complaints are often part of the NCGS clinical picture. Conversely, recent studies support the hypothesis that gluten and other wheat components may trigger IBS symptoms. Due to this overlap and lack of IBS/NCGS biomarkers, and there is a confusing situation for both the primary care physician and the general audience. This review article is aimed to present (1) an update on the complex relationship between NCGS and IBS and (2) the experts’ opinion on this hot topic.

## 2. Methods

In order to provide consensus and promote collaborative research in this field, a group of 23 experts convened in Meran, Italy in December 2016. Each expert was assigned to separate work streams according to their areas of clinical and research experience. Work streams involved review of recent literature using PubMed and Embase Databases. The previous literature review occurred at the Salerno meeting in October 2014. For this reason, the new MeSH-based literature review encompassed all articles since that time but also key historical papers. Furthermore, experts in attendance were also asked to identify emerging studies (presented in abstract form). The experts were then divided into work streams, with sub-section presentations and break-out focus groups. Based on this current evidence, five areas were identified where significant progress within the medical literature has occurred: (1) Prevalence studies of NCGS; (2) Non-IgE wheat allergy; (3) Potentially harmful wheat components; (4) Pathogenetic mechanisms for IBS triggered by wheat; (5) Overlap between IBS-type symptoms and NCGS. This review provides the summary and consensus (undertaken using a Delphi method) from this International Workshop.

### 2.1. Prevalence of NCGS: Emerging Data and Ascertainment Pitfalls

We have previously categorized the spectrum of gluten-related disorders and provided a “road map” for clinicians seeking to manage their patients who have gluten-related symptoms [2,3,4]. Considering the uncertainty regarding the prevalence of NCGS, initial efforts at the Center for Celiac Research, University of Maryland (USA) were aimed at clarifying this with an original report of their experience with 5896 patients seen between 2004 and 2010 [3]. The criteria for NCGS were fulfilled by 347 patients suggesting a prevalence in their study population of 6% (1:17). The limitation to this important observation is that this is a tertiary center seeing patients within a fee-paying system. Due to a selection bias, this may not accurately reflect international prevalence figures for NCGS.

Since that time other international groups have tried to estimate the prevalence of NCGS (Table 1) [5,6,7,8,9,10,11,12,13,14]. Of these studies, three report data from the National Health and Nutrition Examination Survey (2009–2010), with the most recent study incorporating data from 2011 to 2012 [6,7,12]. The range when considering the general population and ensuring exclusion of CD, highlights a prevalence between 0.6% and 10.6%. The huge variability in prevalence figures is mainly explained by lack of diagnostic biomarker(s). In order to clarify the diagnosis of NCGS, at a previous consensus meeting the experts’ group recommended a double-blind placebo controlled (DBPC) approach using 8 g of gluten [4]. Although a DBPC approach is the gold standard for a rigorous scientific identification of true cases, this is difficult to undertake in daily clinical practice. Patients frequently refuse to re-introduce gluten into their diet due to the immediate symptom burden which they perceive. This view is further reflected by the high levels of unsuccessful patient recruitment in studies to resolve these specific issues reported in the literature. There is still very limited data on the overlap between NCGS and IBS-type symptoms. The UK population survey undertaken in 1002 adults demonstrated that individuals with NCGS had an increased prevalence of fulfilling the Rome III criteria for irritable bowel syndrome in comparison with those without NCGS (20% vs. 3.89%, odds ratio 6.23, *p* < 0.0001) [8].

An emerging epidemiological issue is represented by self-reported NCGS, i.e., people excluding gluten-containing food without a medical diagnosis of a specific gluten-related disorder. Many individuals perceive the GFD as healthy life style practice. This group may be termed lifestyles, free from or clean eaters depending on which country they reside in. We propose to define them as people who avoid gluten [5,6,7,8,9,10,11,12,13,14]. These individuals are widely diffused in Western countries, with a prevalence of 6.2–13% [8,13].

They must be treated with great caution and if such patients present to clinicians it is imperative to first exclude celiac disease [2,3,4] or other causes not related to any specific trigger (placebo effect, implementation of a healthier diet, etc.). Such patients can be advised that simply by identifying gluten as a culprit for their symptoms, this gives them a risk ranging from 2% to 42% of having undiagnosed celiac disease (based on the current published literature) [8,15,16,17,18,19]. This extreme variation reflects an ascertainment bias related to the referral patterns of the centers that have published their data. Tertiary and quaternary centers may have a high prevalence of patients presenting with gluten-related symptoms who are subsequently recognized to have celiac disease.

### 2.2. Update on Non-IgE Mediated Wheat Allergy in NCGS 

Wheat allergy is an adverse immune response which occurs reproducibly in affected individuals. The immune mechanism may be IgE or non-IgE mediated. Individuals may have a serological IgE response to wheat but this only demonstrates sensitization. To make a diagnosis of wheat allergy, patients must also describe reproducible symptoms and signs which occur quickly following wheat exposure. Typically, these symptoms may be gastrointestinal, respiratory, at skin level and in some rare cases anaphylaxis or angioedema [20,21]. Recent developments have occurred specifically in the field of adult non-IgE-mediated food allergy. Non-IgE-mediated food allergy is a condition well known by pediatricians who recognize at least three gastrointestinal clinical conditions: the food protein-induced enterocolitis syndrome, the food protein-induced proctocolitis, and the food protein-induced enteropathies [20]. In the pediatric population, this condition is mainly due to cow’s milk protein hypersensitivity (CMPH) but also soya (most common triggers). Wheat is also involved in Food Protein Induced Enteropathy in children. It is difficult to diagnose as radioallergosorbent test or skin testing are neither specific nor sensitive. For this reason, a CMPH diagnosis must be posed exclusively on clinical basis by means of elimination diet and successive DBPC food challenge [22]. It is probable that in adults, the allergy-related gastro-intestinal symptoms are common and often underestimated [21] (confocal/review recent papers).

The hypothesis that NCGS could be a non-IgE-mediated wheat allergy is based on some clinical aspects (presence of a personal history of food allergy in the pediatric age, or of coexistent atopic diseases) [22], laboratory and histological data (positive serum anti-gliadin antibodies, positive cytofluorimetric assay revealing in vitro basophil activation by food antigens, and presence of eosinophils in the intestinal mucosa biopsies) [23,24,25] and by new and exciting endoscopic findings (see below) [26]. Further studies are required to explore the putative allergic mechanisms involved in the pathogenesis of NCGS. Recently immunological activation in the intestinal mucosa of the NCGS patients has been demonstrated. In fact, gastrointestinal food allergies are often mediated by IgE-independent mechanisms involving mast cells, eosinophils, and other immune cells [21]. An increase in mucosal lymphocytes has been reported in a consistent percentage of patients with NCGS diagnosed by DBPC challenge [25,27], and in general most of the studies on NCGS showed that about half of the patients have a raised IELs count >25 per 100 enterocytes [23,28,29]. Very recently, an increased infiltration of innate lymphocytes-1 cells, producing IFN-γ, in the rectal mucosa of NCGS patients has been reported; furthermore, this infiltration decreased after resuming a wheat-free diet [30]. Given these conflicting observations further work is required to clarify whether NCGS could have an association with either IgE or non IgE mediated wheat allergy.

There is a lack of consensus about intestinal permeability in NCGS. After a first report suggesting a reduced intestinal permeability [28], more recent studies have demonstrated an increased permeability in NCGS [31,32]. Uhde et al., in particular, were able to demonstrate a state of systemic immune activation, partly in response to translocated microbial components and possibly in conjunction with a compromised intestinal epithelium, which significantly improved on a wheat-free diet. Further studies are needed to decipher how wheat components can contribute to a compromised gut epithelial barrier and subsequent microbial translocation that result in systemic immune activation.

In this respect, the exciting findings by confocal endomicroscopy could be the keystone. Recently a novel strategy to identify IBS patients with food-related symptoms and specified sensitivities has been described [26]. Within five minutes after the administration of defined food suspensions to the duodenal mucosa of sensitive patients, confocal laser endomicroscopy demonstrated an increase in the number of intraepithelial lymphocytes, epithelial shedding and breaks followed by leaks with secretion of fluorescein into the lumen, and edema with increased inter-villous spaces. Characteristic mucosal changes after administration of a wheat suspension were seen in 13 (and after administration of milk, soy and yeast in another 9) of 36 IBS patients, and in all of the dietary elimination of the identified trigger, including wheat as prominent cause, resulted in a long-term improvement of symptoms. The 36% of wheat-sensitive IBS patients is in line with the proportions revealed in other studies [21,22], and suggests that this method may offer a remarkable sensitivity and specificity for NCGS. A German multi-center study (DRKS00010123) is currently recruiting patients to further validate these findings [33]. Finally, it is worth noting that endomicroscopy with several food challenges is a complex investigation requiring the patient’s sedation.

### 2.3. Potentially Harmful Wheat Components

**Figure 1:**
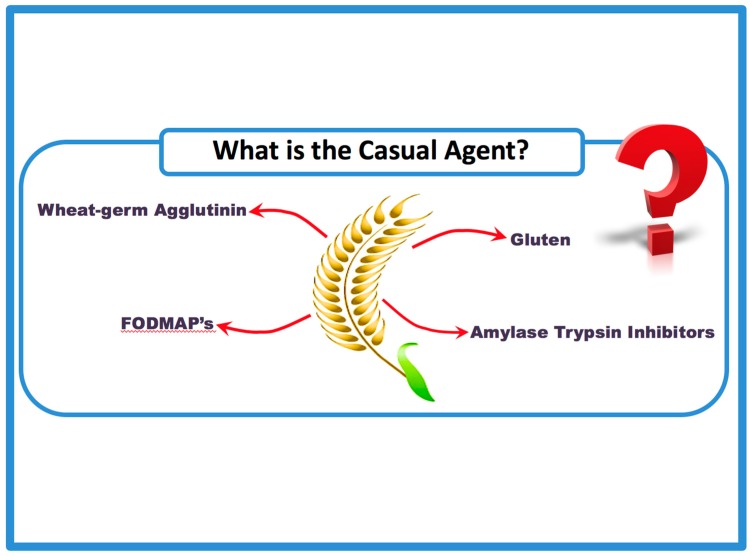
Potential triggers in wheat that may account for both intestinal and extra-intestinal symptoms.

**Figure 2:**
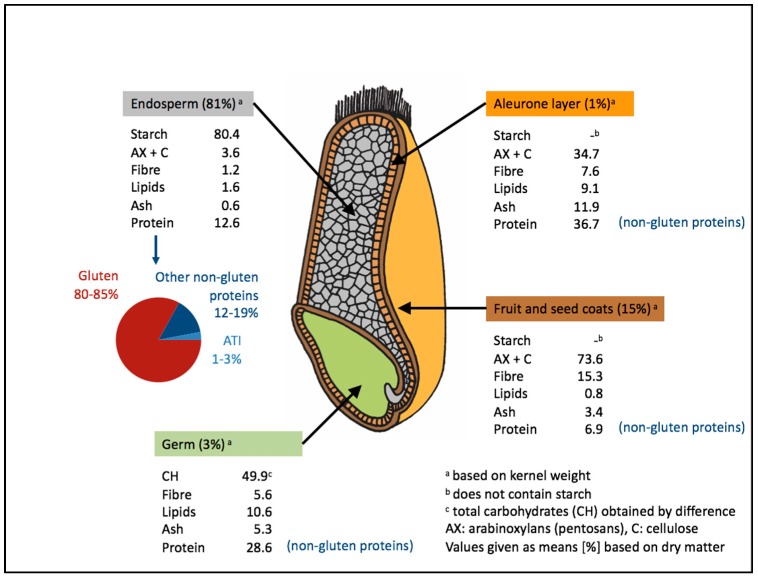
Wheat components.

Several components of wheat are potentially harmful for NCGS and IBS patients including gluten proteins, lipopolysaccharides, amylase/trypsin inhibitors (ATIs), wheat germ agglutinins (WGA) and fermentable oligo-, di-, and monosaccharides and polyols (FODMAPs) (Figure 1) [32,34,35,36,37]. Gluten proteins, the storage proteins of wheat, rye and barley, account for 70–80% of the total grain protein and are located exclusively in the starchy endosperm of the grains (Figure 2). The remaining proteins are albumins and globulins (20–30%) that have metabolic, protective or structural functions. Largely insoluble in water and salt solution, gluten can be subdivided into mostly monomeric prolamins (called gliadins in wheat) soluble in aqueous alcohols and polymeric glutelins (called glutenins in wheat) soluble only in the presence of reducing and disaggregating agents [38]. Specific sequences from gluten, e.g., *N*-terminal peptides from α-gliadin, have been shown to induce an innate immune response in celiac disease [39,40] and could also play a role in NCGS. In general, care is advised when using wheat or gluten for oral challenge, because all wheat is not the same and all gluten is not the same. The composition of the individual components and the overall protein contents can differ in a rather large range depending on wheat species, cultivar, growing conditions (e.g., soil, climate, fertilization) and processing, in the case of gluten and, especially, hydrolyzed gluten. Therefore, each material should be well characterized using proteomic methods and, if possible, standardized [41].

ATIs are a family of at least 11 structurally similar, small and compact mono-, di- or tetrameric wheat proteins, which serve as protective proteins in wheat and other cereals by inhibiting enzymes (amylase and trypsin-like activities) of wheat and some parasites. In the developing grain, ATIs are deposited together with gluten proteins in the endosperm and become associated with the starch granules [42]. Encoded mainly by the B and D genomes, ATIs are high in most modern hexaploid bread wheats, and low in spelt (old hexaploid), tetraploid (durum wheat, emmer) and diploid (einkorn) wheat species. They are also present in other gluten containing cereals such as barley and rye. Long known as major allergens in baker’s asthma [43], ATIs were identified as triggers of innate immune activation in intestinal myeloid cells via stimulation of Toll-like receptor 4 (TLR4) [35]. Notably, nutritional ATIs enhance intestinal inflammation in models of inflammatory bowel disease in mice, and immune activation is higher in the mesenteric lymph nodes than in the intestinal mucosa [44].

Wheat germ agglutinins (WGA) are enriched in the germ of wheat grains with contents from 100 to 500 mg/kg, resulting in typical concentrations of approximately 4 mg/kg in white flour and approximately 30 mg/kg in whole grain flour [37]. Similar to ATIs, WGA are stable against heat and proteolysis and serve as protective proteins. As lectins, WGA are widely recognized as anti-nutrients in foods and bind to glycoproteins, such as human *N*-Acetylneuraminic acid, on the surface of cell membranes. WGA has been shown to induce the release of pro-inflammatory cytokines (TNF-α, IL-1β, IL-12 and IFN-γ) and impair the integrity of the intestinal epithelial layer [45]. However, in contrast to ATIs, no immune stimulatory activity was demonstrated for WGA in vivo.

The term FODMAPs comprises short chain oligosaccharides of fructose (fructans) and galactooligosaccharides (GOS, stachyose, raffinose), disaccharides (lactose), monosaccharides (fructose), and sugar alcohols (polyols), such as sorbitol, mannitol, xylitol and maltitol, that are resistant to digestion and absorption in the human small intestine with complete or partial fermentation in the large intestine. As such, FODMAPs are considered part of dietary fiber (Figure 2). Out of FODMAPs, wheat contains fructans that increase its tolerance to drought and cold. Typical quantities of fructans are 1.5% in white flour and 3.7% in bran [46] and they can be significantly degraded during yeast fermentation (>50%) and almost completely with fermentation [47]. Modern wheat breads contained 0.5–0.7% fructans (based on the product as consumed), spelt bread 0.2% and gluten-free bread 0.2% [48]. However, the fructan content of gluten-free products depends on the specific recipes and the generalized assumption that gluten-free products are always lower in FODMAPs compared to their wheat-containing counterparts is questionable, because e.g., corn flakes and rice bubbles each had 1.1% fructan [48], and another study did not find a significant difference between various wheat breads and gluten-free breads, because the average fructan content was approximately 1% in both [49].

### 2.4. Pathogenetic Mechanisms of IBS in Which Wheat can be the Trigger

Growing evidence indicates that the majority of IBS patients report a significant worsening of symptoms, e.g., abdominal pain, bloating and bowel habit abnormalities, after meals, while experiencing an improvement during fasting [50,51]. Furthermore, some IBS patients clearly identify foods that are thought to be more offending as they evoke more commonly the aforementioned digestive symptoms. Although the relationship between food ingestion and symptom generation in IBS patients has long been established, our understanding of dietary triggers and pathogenic mechanisms involved in IBS is still poorly defined [52]. Wheat is regarded as a possible culprit of symptom generation in some cases of IBS [53]. Wheat administered via endoscopic probe into the duodenal mucosa, was able to alter the integrity of the small intestinal mucosa as shown by epithelial leaks/gaps and widened intervillous spaces confocal detected by using laser endomicroscopy. Taken together both endomicroscopic features and histopathological abnormalities provide a morphological basis to the existence of (inflammatory) wheat sensitivity at least in a substantial subset of patients with IBS [26].

The insufficient degradation of gluten and certain other wheat proteins by small intestinal proteases leaves undigested peptides that can pass through a more permeable epithelial barrier (the so called “leaky gut”), reach the submucosa and activate the resident innate immune cells. Experimental data demonstrated that human leucocyte antigen DQ8 (HLA-DQ8) transgenic mice sensitized by gluten show an altered barrier function and enhanced muscle contractility (likely via an increased release of the excitatory transmitter acetylcholine from myenteric neurons), thereby mimicking mechanisms which are known to occur in patients with IBS. Both enhanced intestinal permeability and increased smooth muscle contractility reverted to normal after gluten withdrawal [54]. A recent clinical trial in patients with IBS with predominant diarrhea (IBS-D) confirmed the close relationship between wheat-containing food and a subgroup of IBS cases [55]. Withdrawal of wheat led to a significant improvement of intestinal symptoms together with a reduced small intestinal permeability, whereas wheat-containing food challenge was followed by the recurrence of symptoms and altered barrier function. These changes were linked to HLA-DQ2/8-positive cases. Markers of intestinal permeability such as small intestinal expression of myosin light chain kinase activity and enhanced colonocyte expression of claudin-15 significantly increased after the challenge and decreased following wheat withdrawal. Furthermore, in addition to gluten-derived peptides, both ATIs and WGA can trigger innate immune pathways.

ATIs elicit innate immune responses in vitro and in vivo, driving intestinal inflammation through the activation of TLR4, as exemplified by the worsening of intestinal inflammation in models of IBD [35,44]. In vitro WGA induces inflammatory response by immune cells leading to the release of IL-4 and IL-13 cytokines from human basophils, and can impair the integrity of intestinal barrier by increasing small intestinal permeability [37]. In addition to proteins, wheat contains fructans that also have a role in IBS symptoms. These are carbohydrates belonging to FODMAPs, which exert an important role in gut homeostasis by modulating the microbiota composition and short chain fatty acid synthesis. Fructans are also contained in fruits, vegetables and dairy products. In IBS, patient symptoms such as bloating, abdominal pain and altered bowel habit may have an association with enhanced bacterial colonic fermentation and gas production in the gut lumen. As a result, luminal distension occurs and through this an activation of enteric mechanoreceptors which generate IBS symptoms. However, it is worthy to mention that FODMAPs do not trigger extra-intestinal (e.g., neurological and fibromyalgia-like) manifestations commonly observed in NCGS, likely elicited by wheat proteins (i.e., gluten, ATIs and possibly WGA). Moreover, FODMAPs are generally considered beneficial by promoting intestinal (epithelial) integrity and health.

Regardless of the identification of the offending components, the scientific community agrees that the withdrawal of wheat from the diet can significantly improve symptoms in a subset of IBS patients, who can sometimes be diagnosed as NCGS. The group of IBS patients with gluten sensitivity gathers the vast majority of wheat sensitive patients, since only a minority of NCGS cases do not display a coexistent IBS [56]. The interplay between the various components of wheat, such as gluten, ATIs, WGA and FODMAPs, may elicit a wide array of both intestinal and extraintestinal symptoms in a subgroup of IBS patients by modulating intestinal permeability, microbiota composition, immune activation which, alone or more likely in combination, also affect the gut-brain axis activity thereby leading to symptom perception. Future research is awaited to decipher the complex interplay between food and digestive sensory-motor function in IBS and allied food-related disorders.

### 2.5. Overlap between IBS-Type Symptoms and NCGS

The prevalence of IBS globally has been estimated to be between 10% and 20% [57,58]. Approximately 50% of patients with gastrointestinal complaints seen in primary care have IBS-type symptoms [59]. Patients with IBS report a reduced quality of life and there is an associated economic and societal cost [60,61]. It is recommended that clinicians should make a positive diagnosis of IBS based on clinical features alone, currently using the Rome IV criteria [62]. The Rome IV criteria categorize IBS by the most predominant presenting symptom-diarrhea (IBS-D), constipation (IBS-C), mixed (IBS-M) or unspecified (IBS-U).

Patients have always reported that food plays an important role in their IBS-type symptoms with estimates of up to 80% of patients having postprandial symptomology, and up to 40% reporting specific “food intolerances” [63,64,65]. Historically, successful restrictive diets for IBS symptoms have been reported by specific research groups. This work has never been translated into daily clinical practice. However, over the last 10 years there has been renewed interest in the concept of dietary interventions for functional gastrointestinal disorders [66,67].

IBS dietary research has focused on the role of two common components of the western diet, specifically FODMAPs and gluten in relation to the induction of IBS symptoms (Figure 1). Initially a retrospective audit evaluated the effect of a low-fructose/fructan diet demonstrating symptomatic relief in 74% of participants with IBS symptoms [68]. Since then, several randomized control trials (RCTs) have demonstrated the efficacy of the low FODMAP diet (LFD) and probable mechanisms. A recent meta-analysis of six RCTs showed that a LFD lead to a 56% greater likelihood of decreasing IBS-SSS (symptom severity scores), however the analysis was limited by information on adherence figures and numerical quantities of FODMAPs ingested [69,70].

The LFD is a complex diet requiring delivery by an experienced dietitian to help ensure both success and overall nutritional adequacy [57,70,71]. The implementation of a low FODMAP diet, involves a strict reduction of all FODMAP groups for 4–8 weeks. This is then followed by a re-introduction of one FODMAP group per week, as tolerated by the patient. The drastic reduction of FODMAP intake could have negative physiological consequences on the colonocyte metabolism, the intestinal microbiota, and the nutritional status, which need further investigation. A significant reduction in iron and calcium intakes during a four-week trial (elimination phase of the LFD) has been reported [71,72,73]. Another potential problem is that the public may commence a LFD without an appropriate reintroduction phase under the guidance of a specialist dietitian. This is of concern considering that the LFD alters the colonic microbiota. A significant reduction in bifidobacteria after four weeks of a LFD has been observed [74]. A recent Australian trial showed that FODMAP restriction was associated with a higher fecal pH, significantly lower bacterial load and diversity, and a significant reduction in colonic bacterial groups with known health benefits when compared to the typical Australian diet [75]. Both studies suggest further trials are required to assess the long-term effects of FODMAP reduction on the microbiome and overall health.

Dietary adherence is another predictor of response in the LFD that warrants further investigation. Dietetic guidance and supportive written (or digital) information are essential. However, even with these aspects in place, the initial exclusion and reintroduction phases can be difficult to follow. Not all the LFD studies have reported a beneficial outcome for IBS patients and first line dietary advice has also been reported as of similar efficacy to FODMAP reduction [76,77,78,79].

There is overlap between NCGS and IBS-type symptoms [8,10]. The fundamental difference between NCGS and IBS is that patients with NCGS self-report symptoms when consuming gluten and have identified or perceive gluten as the culprit. Conversely IBS patients do not report gluten as a specific stimulus for their symptoms. However, previously published literature has demonstrated that wheat is a commonly reported “food intolerance” when IBS patients are specifically questioned [63,64,65]. There is now a body of literature supporting the role of a GFD as a specific dietary intervention in IBS (Table 2). The mechanism by which wheat or specific wheat components such as gluten or ATIs cause IBS-type symptoms is debated. Wheat (and even gluten) contains a number of compounds, apart from gluten, that could produce a symptomatic response; perhaps the most obvious of these are FODMAPS, specifically fructans (Figure 1 and Figure 2). One trial showed that individuals with self-reported NCGS (and IBS-type symptoms) already on a GFD further benefited when placed on a low FODMAP diet and found no specific or dose-dependent effect of gluten [80]. However, the participants reported very high visual analogue scale ratings for their symptoms at the start of the study when they were already on a GFD. This is unlikely to be representative of the NCGS population. Furthermore, this study’s double-blind placebo-controlled crossover design, where all participants cycled through high-dose, low-dose or no gluten control diets could have produced an anticipatory nocebo response [81]. It is also interesting to note that all the participants returned to a GFD at the end of the trial as they “subjectively described feeling better” [82]. Research into the GFD in the treatment of IBS-D is summarized in Table 2.

**Figure 3:**
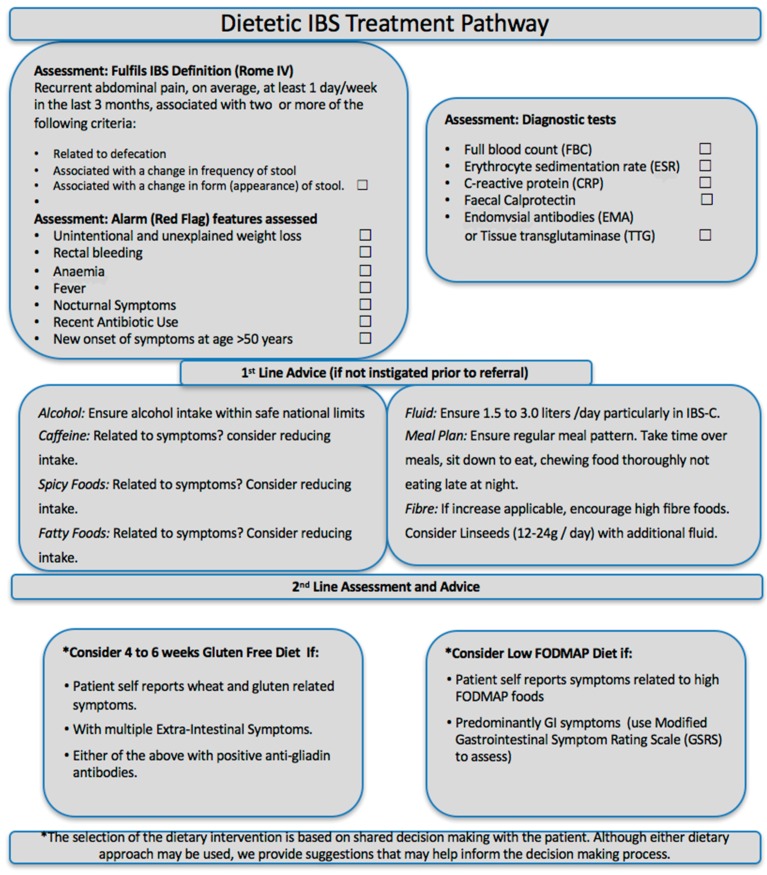
IBS treatment pathway.

Based on current evidence a gluten (and thus wheat) free diet also appears to be a potential dietary intervention for a subgroup of IBS patients and the term of gluten sensitive IBS has been coined. Like a LFD, a GFD has been shown to cause a reduction of lacto and bifidobacteria in the gut. There are also some studies suggesting lower intake of calcium, iron, folate and fiber, when compared to a gluten-containing diet [95,96,97,98]. There is clearly overlap between a GFD and a LFD. One of the key excluded components of a LFD is wheat; furthermore, patients on a long-term LFD (following the reintroduction phase) appear to view the reduced intake of wheat as essential to their maintained symptomatic response [99]. Thus, a GFD may be a more practical option for IBS patients, which does not impair their food-related quality of life to the same level as a LFD (Figure 3).

## 3. Conclusions

Since our initial consensus document of 2012 there has been a significant expansion of the published data in the field of gluten-related disorders. We now have an appreciation of two groups of patients who may benefit from a GFD, firstly those who present with self-reported gluten-related symptoms and may have NCGS and secondly those who present with IBS-type symptoms and could have gluten or wheat sensitive IBS. Clinicians involved in the management of IBS now have an exciting range of dietary interventions that may benefit their patients (Figure 3).

## Figures and Tables

**Table 1:** Prevalence studies of people avoiding gluten-based products.

Author	Year of Publication	Country	Group	Sample Size	Avoidance of Gluten-Based Products	Known Previous Diagnosis of CD
Tanpowpong et al. [5]	2012	New Zealand	Children-general population	916	5% (*n* = 48)	1% (*n* = 9)
Rubio-Tapia et al. [6]	2013	USA	Age ≥ 6 years, National Health and Nutrition Examination Survey (NHANES) 2009–2010	7798	0.63% (*n* = 55)	0.1% (*n* = 6)
DiGiacomo et al. [7]	2013	USA	NHANES	7762	0.6%	
Aziz et al. [8]	2014	UK	Adults-general population	1002	3.7% (*n* = 37)	0.8% (*n* = 8)
Lis et al. [9]	2014	Australia	Adults-athletes	910	41.2% (*n* = 375)	None
Volta et al. [10]	2014	Italy	Adult and pediatric GI clinic population	12,225	391 (3.2%)	Within same population 340 (2.8%) coeliac patients diagnosed
Golley et al. [11]	2015	Australia	Adults-general population	1184	10.6% (*n* = 126)	1.2% (*n* = 14)
Mardini et al. [12]	2015	USA	Age ≥ 6 years, NHANES 2009–2010 & 2011–2012 data combined	14,701	0.9% (*n* = 142)	0.1% (*n* = 21)
Van Gils T et al. [13]	2016	The Netherlands	Adults-general population	785	6.2% (49/785)	0.25% (2/785)
Carroccio A et al. [14]	2017	Italy	Age 14–18	548	2.9% (16/548)	1.26% (7/555)

**Table 2:** Summary of studies examining the role of gluten and wheat in IBS.

Lead Author	Country	Year	Patients	Outcome
Wahnschaffe [83]	Germany	2001	102 IBS-D without CD	Stool frequency significantly improved in patients HLA DQ2/DQ8 + ve
Wahnschaffe [84]	Germany	2007	145 IBS-D without CD	HLA-DQ2 predicted response to GFD
Biesikierski [85]	Australia	2010	34 NCGWS	Significant reduction in symptoms in GFD group
Carroccio [25]	Italy	2012	920 patients with IBS	70 patients wheat-sensitive and 206 food sensitivities
Vazquez-Roque [86]	USA	2012	45 patients with IBS-D	Increased intestinal permeability in patients receiving gluten
Vazquez-Roque [87]	USA	2013	45 patients with IBS-D	Reduction in stool frequency in patients on GFD
Biesikierski [80]	Australia	2013	37 NCGWS on GFD	Patients responded to reduction in FODMAPs during run-in but no difference between GFD and gluten-containing arms
Fritscher-Ravens [26]	Germany	2014	36 patients with food-sensitive IBS 13/36 GFD after positive wheat challenge in CLE	All patients improved significantly on the GFD for at least one year
Aziz [88]	UK	2015	40 patients with IBS-D	70% had reduced symptomology with GFD for 6 weeks
Di Sabatino [89]	Italy	2015	59 self-reported NCGWS	4 g of gluten per day for 1 week increased overall clinical symptoms compared with placebo in (*p* = 0.034)
Shahbazkhani [90]	Iran	2015	72 patients with IBS (Based on Rome III criteria)	Worsening of intestinal symptoms with gluten compared to placebo
Zanini [91]	Italy	2015	35 NCGWS on a GFD	Given either and containing or gluten-free flour. 34% symptomatic with gluten-containing flour, 49% symptomatic with gluten-free flour, 17% no response
Zanwar [92]	India	2016	60 patients with IBS (Based on Rome III criteria)	GFD for 4 weeks. Significant reduction in visual analogue scales (VAS) of symptomology
Elli [93]	Italy	2016	140 patients enrolled	14% of patients shown to have symptomatic response to gluten on repeat challenge
Barmeyer [94]	Germany	2017	34 patients with IBS	34% responded to a GFD and continued on a GFD at 1 year
